# Reply to: Oligoclonality of TRBC1 and TRBC2 in T cell lymphomas as mechanism of primary resistance to TRBC-directed CAR T cell therapies

**DOI:** 10.1038/s41467-025-56396-7

**Published:** 2025-01-29

**Authors:** Mathieu Ferrari, Farhaan Parekh, Paul Maciocia, Pedro Horna, Simon Thomas, Andrew K. Sewell, Martin Pule

**Affiliations:** 1 Research Division, Autolus Therapeutics, London, UK; 2 Research Department of Haematology, University College London, London, UK; 3 Laboratory Medicine and Pathology, Mayo Clinic, Rochester, MN USA; 4 Division of Infection and Immunity, Cardiff University School of Medicine, Cardiff, UK; 5 Systems Immunity Research Institute, Cardiff University, Cardiff, UK

**Keywords:** Immunotherapy, T-cell lymphoma, Tumour heterogeneity, T cells

## Introduction and results

**replying to** B. Thiele et al. *Nature Communications* 10.1038/s41467-025-56395-8 (2025)

Targeting pan T cell antigens to treat T cell lymphomas (TCLs) risks profound immunosuppression caused by concomitant depletion of the entire T cell compartment. Recently, we proposed exploiting TCRβ gene rearrangement to more safely target T cell lymphomas^1^. TCRαβ T cells irreversibly select a TCRβ constant chain from either of the two isoforms TRBC1 or TRBC2 due to allelic exclusion during TCR gene re-arrangement. During malignant transformation, assuming the malignancy arose from a single mature T cell, the resulting lymphoma should homogenously and exclusively express only one TRBC chain. Since normal TCRαβ T cells comprise of a mixture of either TRBC1 or TRBC2 T cells, this allows for selective targeting of TRBC isoform expressed by the lymphoma, with preservation of normal T cells which express the alternate isoform^2,3^.

Nearly all TCLs express TCRαβ on the cell surface, thus TRBC1/TRBC2 targeting is an attractive therapeutic strategy^4–7^. In our original paper^1^, we explored a TRBC1 directed therapeutic. In the arising paper, we described the structural basis for antibody discrimination between TRBC1 and 2 and described a TRBC2 directed therapy^8^. Additionally, we have recently published early data from an ongoing phase I/II clinical study evaluating TRBC1 targeted autologous chimeric antigen receptor (CAR) T cells (NCT03590574) in patients with relapsed/refractory TRBC1^+^ T cell lymphoma^9^. At the highest dose-level, 3/4 patients achieved complete metabolic response, with 2/4 in ongoing remission past the 18-month mark. Relapse or resistance with TCRαβ negative or TRBC isoform switched disease was not observed, but poor CAR T cell persistence may be a limitation of the CAR T cell approach^10^.

**Fig. 1:**
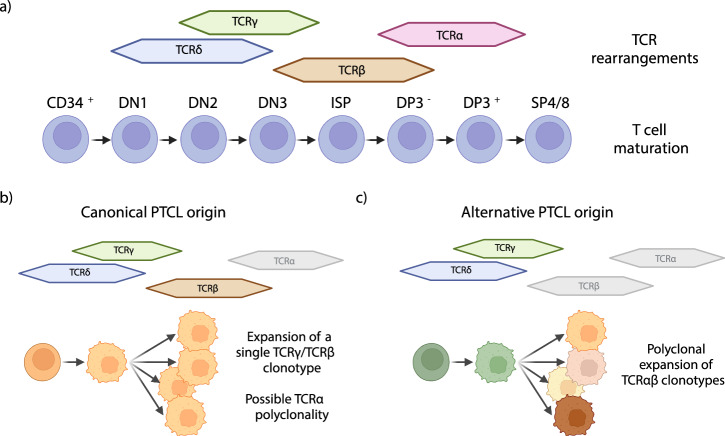
PTCL origin. **a** Schematic representation of T cell maturation and TCR rearrangements. **b** Canonical mature TCLs origin from progenitor cells that have already performed TCRδ/γ/β rearrangements, displaying a single TCRβ clonotype. **c** Alternative mature TCL origin proposed by Thiele et al., derived from immature progenitor cells not committed on TCRβ and displaying polyclonal TCRαβ clonotypes. Created in BioRender. Neves, M. (2024) https://BioRender.com/z67h786.

In the matters arising, Thiele et al. challenge our targeting approach by suggesting that rearrangement of the TCRβ locus continues after malignant transformation. In line with this, Iyer et al. recently challenged the mature T cell origin hypothesis of PTCL^11^ (Fig. 1b). T cells undergo sequential rearrangement of TCR V(D)J gene loci, starting with δ, followed by γ, β, and α (Fig. 1a). Malignant T cells may have clonal V(D)J rearrangement of earlier chains but may retain potential for V(D)J rearrangement for loci not yet rearranged at the point of transformation (Fig. 1c). Iyer’s challenge was based on analysis of ES, whole genome sequencing (WGS), and whole-transcriptome sequencing (WTS) from 574 cases of peripheral T cell lymphomas (PTCL). Iyer found that while most cases appeared clonal for TCRγ, in nearly all cases, multiple β clones were detected. In the matters arising, Thiele et al. study scRNA-seq data from 12 TCL cases. They found that 7/12 cases had polyclonal TCRβ expression and 2/12 lacked TCRβ expression.

A more complex origin hypothesis for PTCLs contradicts current practise which assumes that TCLs are derived from a mature T cell. In fact, diagnosis of TCL often uses clonotypic assessment of TCRγ and TCRβ using multiplexed PCR amplification^12,13^. Clonality of Vβ is often used for MRD in T-ALL^14^, and most PTCL and other mature TCL lack expression of the terminal deoxynucleotidyl transferase^15^. Notably, at a protein level, in our immunohistochemical studies^1^, and more recent flow-cytometric studies, most cases predominantly and homogeneously express one or the other TRBC isoform^2,3^. Thiele challenges our flow-cytometric findings by noting the presence of small numbers of apparently non-clonal cells within a malignant population. However, flow cytometry gating strategies are imperfect due to immunophenotypic overlap between tumour cells and reactive T cells, and these findings are not unexpected^16^.

The apparent TCRβ genomic diversity found by Iyer et al. and Thiele et al. has alternative explanations. Firstly, unproductive rearrangement of the excluded TCRβ locus is common, and WGS/ WES do not discriminate productive and non-productive chains. Further, WGS and WES often result in low sequencing depth for the TCR region, leading to high noise unless validated by other methods^17^. Similarly, WTS datasets, such as the one used in Iyer et al.^18^, suffer from low coverage, impacting data quality and reliability^19^.

**Fig. 2:**
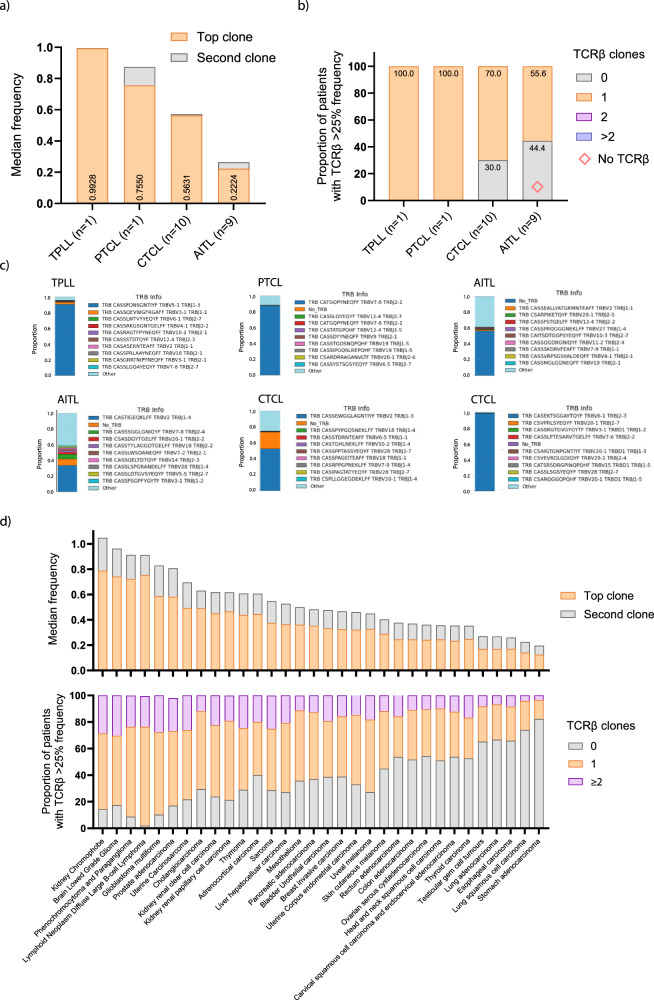
TCRβ clonotype frequency. **a** Median frequency of the top 2 TCRβ clonotypes in AITL, CTCL, PTCL and TPLL cohorts (Thiele et al., Liu et al.^20^, Suma et al. ^21^). **b** Proportion of patients with TCRβ clonotypes above 25% frequency threshold in AITL, CTCL, PTCL and T-PLL cohorts (Thiele et al., Liu et al. ^20^, Suma et al. ^21^). Grey bars indicate % of patients for each indication showing no TCRβ clonotype above 25% threshold. Orange bars indicate 1 TCRβ clonotype above the threshold, purple bars indicate 2 TCRβ clonotypes above the threshold, and blue bars indicate over 2 TCRβ clonotypes above the threshold. Diamond indicates % of patients with TCRβ negative cells above the threshold. **c** Representative TCRβ clonotype diversity in selected patients (top 10 TCRβ clonotypes reported). **d** Pan cancer scRNA seq dataset^22^ screened for top 2 TCRβ clonotype median frequency (top) and proportion of patients with TCRβ clonotypes above 25% frequency threshold. Grey bars indicate % of patients for each indication showing no TCRβ clonotype above 25% threshold. Orange bars indicate 1 TCRβ clonotype above the threshold, purple bars indicate 2 or more TCRβ clonotypes above the threshold.

We reanalysed the TCRβ clonotype frequency in the patients’ samples analysed by Thiele et al. using sequencing data from Liu et al.^20^and Suma et al.^21^. We quantified the dominant TCRβ clonotype in each sample and found a median frequency of 22.2% in Angioimmunoblastic T-cell lymphoma (AITL) (*n* = 9, range 4.4–100), 56.3% in Cutaneous T-cell lymphoma (CTCL) (*n* = 10, range 1.2–98.3), 75.5% in PTCL (*n* = 1), and 99.3% in T-Cell Prolymphocytic Leukemia (TPLL) (*n* = 1). The second most abundant TCRβ clonotype accounted for median frequencies of 4.1%, 0.8%, 11.8% and 0.1% in AITL, CTCL, PTCL, and TPLL, respectively (Fig. 2a). Application of the 25% threshold for clonotypic expansion set by Iyer et al.^11^, resulted in a predominant single clone expansion in 14 out of 21 samples, with 7 out of 21 showing no TCRβ chain above threshold. Interestingly, one AITL sample showed a predominant expansion of a TCRβ-negative clone (Fig. 2b). The top 10 TCRβ clonotypes for representative samples are shown in Fig. 2c.

The challenge with this analysis is determining whether the TCRβ rearranged cells falling outside expression of the dominant Vβ are malignant or derived from contaminating normal T cells, which may also be clonally expanding. In this light, the 5% threshold adopted by Thiele et al. is likely too low and even the arbitrary 25% threshold proposed by Iyer et al. may not exclude infiltrating normal T cells. For instance, we analysed a dataset of non-T cell lymphoid malignancies^22^, finding a median top TCRβ clone frequency of 24.6% (*n* = 6402, range 0.9–100), with 20 out of 32 indications showing a predominant single T cell clonal expansion (Fig. 2d). This highlights the high degree of reactive T cell infiltrates that can be expected in malignant biopsies.

Without the ability to correlate TCRβ data with TCRγ sequencing and transcriptomic profiles, some of the divergent TCRβ clonotypes identified may relate to expanded infiltrating healthy T cells responding to tissue or tumour challenges. Since Thiele et al., did not provide such analysis, we believe their findings do not add significantly to what has already been described by Iyer et al.^11^.

In summary, technical limitations and small numbers of samples limit the usefulness of the findings of Thiele et al. However, the elegant work of Iyer does indicate that the clonal origin of PTCL is likely complex. The degree of re-arrangement of the TCRβ locus may vary with different PTCL lymphoma subtypes and as Iyer et al. suggested, clonal evolution at a malignant cell level may further influence TCRβ usage. A detailed prospective analysis of TRBC1/2 surface protein expression in a large set of PTCLs coupled with methods which allow discrimination from normal T cells is needed to determine how widely useful TRBC1/2 targeting will be. We hope that the selective TRBC2 antibody described in this present paper will facilitate such studies.

## Methods

T-cell receptor (TCR) repertoire data were retrieved from publicly available data repositories (Thiele et al., Suma et al.^21^, Liu et al.^20^). Where available, original Cell Ranger outputs were utilised; otherwise, raw data were reprocessed using Cell Ranger 8.0.1 VDJ pipeline with the Human reference (GRCh38/Ensembl/10x). Alpha-beta clonotype thresholding was performed using Cell Ranger-derived clonotypes. TCRs were classified as clonally enriched when present in proportions exceeding a specified threshold. To account for potential ongoing alpha chain rearrangement, we also analysed aggregated beta clonotypes, combining all TCRs sharing beta chains to determine beta chain utilisation.

Pan cancer dataset from The Cancer Genome Atlas (TCGA)^22^ was used to investigate the top TCRβ clonotypes across a broad spectrum of non-T-cell cancers. For each patient, it was calculated the median frequency of the top 2 TCRβ clonotypes and the proportion of patients with TCRβ clonotypes exceeding a specified threshold.

Data analysis and plotting performed with GraphPad Prism v 10.1.2. Illustrations for Fig. 1 were done with BioRender (https://BioRender.com).

### Reporting summary

Further information on research design is available in the Nature Portfolio Reporting Summary linked to this article.

## Supplementary information

Reporting Summary

## Source data

Source Data

## Data Availability

Source data are provided as Source data File. For data by Thiele et al., Suma et al.^21^, Liu et al.^20^, and Thorsson et al.^22^, please refer to their respective publications. Source data are provided with this paper.
