# Supplementary material for: Reply to: Oligoclonality of TRBC1 and TRBC2 in T cell lymphomas as mechanism of primary resistance to TRBC-directed CAR T cell therapies
Source: Nat Commun. 2025 Jan 29;16:1103. doi: 10.1038/s41467-025-56396-7 (PMC11779960; doi:10.1038/s41467-025-56396-7)
Supplement: Supplementary file 1 — Reporting Summary [file 41467_2025_56396_MOESM1_ESM.pdf]

nature portfolio

Corresponding author(s): Martin Pule

Last updated by author(s): Dec 20, 2024

Reporting Summary

Nature Portfolio wishes to improve the reproducibility of the work that we publish. This form provides structure for consistency and transparency in reporting. For further information on Nature Portfolio policies, see our Editorial Policies and the Editorial Policy Checklist.

Statistics

For all statistical analyses, confirm that the following items are present in the figure legend, table legend, main text, or Methods section.

n/a	Confirmed	
☐	☒	The exact sample size (*n*) for each experimental group/condition, given as a discrete number and unit of measurement
☒	☐	A statement on whether measurements were taken from distinct samples or whether the same sample was measured repeatedly
☒	☐	The statistical test(s) used AND whether they are one- or two-sided<br>*Only common tests should be described solely by name; describe more complex techniques in the Methods section.*
☒	☐	A description of all covariates tested
☒	☐	A description of any assumptions or corrections, such as tests of normality and adjustment for multiple comparisons
☒	☐	A full description of the statistical parameters including central tendency (e.g. means) or other basic estimates (e.g. regression coefficient) AND variation (e.g. standard deviation) or associated estimates of uncertainty (e.g. confidence intervals)
☒	☐	For null hypothesis testing, the test statistic (e.g. *F*, *t*, *r*) with confidence intervals, effect sizes, degrees of freedom and *P* value noted<br>*Give P values as exact values whenever suitable.*
☒	☐	For Bayesian analysis, information on the choice of priors and Markov chain Monte Carlo settings
☒	☐	For hierarchical and complex designs, identification of the appropriate level for tests and full reporting of outcomes
☒	☐	Estimates of effect sizes (e.g. Cohen's *d*, Pearson's *r*), indicating how they were calculated

Our web collection on statistics for biologists contains articles on many of the points above.

Software and code

Policy information about availability of computer code

Data collection	T-cell receptor (TCR) repertoire data were retrieved from publicly available data repositories (Thiele et al. Matters arising letter, Suma et al. Leukemia 2024, Liu et al. Nature communications 2022). Pan cancer dataset from The Cancer Genome Atlas (TCGA) (Thorsson et al., Immunity 2018) was used to investigate the top TCRβ clonotypes across a broad spectrum of non-T-cell cancers
Data analysis	Where available, original Cell Ranger outputs were utilized; otherwise, raw data were reprocessed using Cell Ranger 8.0.1 VDJ pipeline with the Human reference (GRCh38/Ensembl/10x). Alpha-beta clonotype thresholding was performed using Cell Ranger-derived clonotypes. TCRs were classified as clonally enriched when present in proportions exceeding a specified threshold. To account for potential ongoing alpha chain rearrangement, we also analysed aggregated beta clonotypes, combining all TCRs sharing beta chains to determine beta chain utilization.<br>For Pan Cancer dataset, for each patient, it was calculated the median frequency of the top 2 TCRβ clonotypes and the proportion of patients with TCRβ clonotypes exceeding a specified threshold.

For manuscripts utilizing custom algorithms or software that are central to the research but not yet described in published literature, software must be made available to editors and reviewers. We strongly encourage code deposition in a community repository (e.g. GitHub). See the Nature Portfolio guidelines for submitting code & software for further information.

Data

Policy information about availability of data

All manuscripts must include a data availability statement. This statement should provide the following information, where applicable:

- Accession codes, unique identifiers, or web links for publicly available datasets
- A description of any restrictions on data availability
- For clinical datasets or third party data, please ensure that the statement adheres to our policy

Source data are provided as Source data File.
For data by Thiele et al., Suma et al., Liu et al., and Thorsson et al., please refer to their respective publications.

Research involving human participants, their data, or biological material

Policy information about studies with human participants or human data. See also policy information about sex, gender (identity/presentation), and sexual orientation and race, ethnicity and racism.

Reporting on sex and gender	Sex and gender of patients data used in this manuscript were not available to researchers.
Reporting on race, ethnicity, or other socially relevant groupings	Race and ethnicity of patients included in this manuscript were not available to researchers.
Population characteristics	Patients malignant disease condition is reported in Figure 2. No additional information was available to researchers
Recruitment	Data was obtained from publicly available repositories from Thiele et al. Matters arising letter, Suma et al. Leukemia 2024, Liu et al. Nature communications 2022, and Thorsson et al. Immunity 2018.
Ethics oversight	Data was processed and analysed according to Autolus procedures (according to Human Tissue Authority licence 12642, UK)

Note that full information on the approval of the study protocol must also be provided in the manuscript.

Field-specific reporting

Please select the one below that is the best fit for your research. If you are not sure, read the appropriate sections before making your selection.

☒ Life sciences ☐ Behavioural & social sciences ☐ Ecological, evolutionary & environmental sciences

For a reference copy of the document with all sections, see nature.com/documents/nr-reporting-summary-flat.pdf

Life sciences study design

All studies must disclose on these points even when the disclosure is negative.

Sample size	Included in the study are 9 AITL, 10 CTCL, 1 PTCL, 1 TPLL and 6402 non T-cell malignancies
Data exclusions	Patients malignant samples derived from peripheral blood biopsies were excluded from the analysis
Replication	Not applicable
Randomization	Not applicable
Blinding	Not applicable

Reporting for specific materials, systems and methods

We require information from authors about some types of materials, experimental systems and methods used in many studies. Here, indicate whether each material, system or method listed is relevant to your study. If you are not sure if a list item applies to your research, read the appropriate section before selecting a response.

Materials & experimental systems

n/a	Involved in the study	
☒	☐	Antibodies
☒	☐	Eukaryotic cell lines
☒	☐	Palaeontology and archaeology
☒	☐	Animals and other organisms
☐	☒	Clinical data
☒	☐	Dual use research of concern
☒	☐	Plants

Methods

n/a	Involved in the study	
☒	☐	ChIP-seq
☒	☐	Flow cytometry
☒	☐	MRI-based neuroimaging

Clinical data

Policy information about clinical studies

All manuscripts should comply with the ICMJE guidelines for publication of clinical research and a completed CONSORT checklist must be included with all submissions.

Clinical trial registration: Not applicable

Study protocol: Not applicable

Data collection: AITL, CTCL, PTCL and TPLL cohorts were obtained from publicly available datasets reported by Thiele et al. Matters arising letter, Suma et al. Leukemia 2024, Liu et al. Nature communications 2022. Pan cancer dataset was obtained from The Cancer Genome Atlas (TCGA) (Thorsson et al. Immunity 2018)

Outcomes: Not applicable

Plants

Seed stocks: Not applicable

Novel plant genotypes: Not applicable

Authentication: Not applicable
